# Comparison of structural dynamics and coherence of d–d and MLCT light-induced spin state trapping†
†Electronic supplementary information (ESI) available. See DOI: 10.1039/c6sc05624e



**DOI:** 10.1039/c6sc05624e

**Published:** 2017-04-24

**Authors:** S. Zerdane, L. Wilbraham, M. Cammarata, O. Iasco, E. Rivière, M.-L. Boillot, I. Ciofini, E. Collet

**Affiliations:** a Univ Rennes 1 , CNRS , Institut de Physique de Rennes , UMR 6251 , UBL , F-35042 Rennes , France . Email: marco.cammarata@univ-rennes1.fr ; Email: eric.collet@univ-rennes1.fr; b Institut de Recherche de Chimie Paris , PSL Research University , CNRS , Chimie ParisTech , 11 Rue Pierre et Marie Curie , F-75005 Paris , France; c Univ Paris Sud , Université Paris-Saclay , CNRS , Institut de Chimie Moléculaire et des Matériaux d'Orsay , UMR 8182 , Orsay , France

## Abstract

Light-induced excited spin state trapping (LIESST) in Fe^II^ spin-crossover systems is a process that involves the switching of molecules from low (LS, *S* = 0) to high spin (HS, *S* = 2) states. The direct LS-to-HS conversion is forbidden by selection rules, and LIESST involves intermediate states such as ^1,3^MLCT or ^1,3^T. The intersystem crossing sequence results in an HS state, structurally trapped by metal–ligand bond elongation through the coherent activation and damping of molecular breathing. The ultrafast dynamics of this process has been investigated in FeN_6_ ligand field systems, under MLCT excitation. Herein, we studied LIESST in an Fe^II^N_4_O_2_ spin-crossover material of lower symmetry, which allowed for quite intense and low-energy shifted d–d bands. By combining *ab initio* DFT and TD-DFT calculations and fs optical absorption measurements, we demonstrated that shorter intermediates enhanced coherent structural dynamics, and d–d excitation induced faster LS-to-HS switching, compared to MLCT.

## Abstract 2

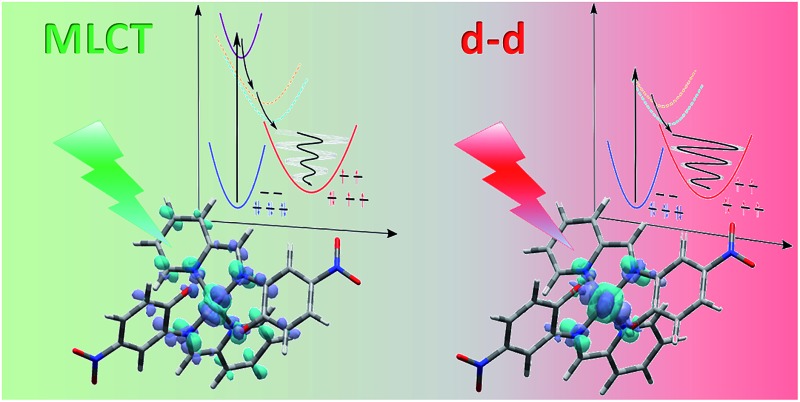
Femtosecond optical spectroscopy revealed that the coherent structural dynamics accompanying LIESST was stronger with d–d than MLCT excitation.

## Introduction

The development of photochemistry and photophysics of coordination compounds requires a good understanding the role of intermediate excited states.^1^ Light-induced excited spin state trapping (LIESST) is a process that is able to switch molecules between different spin states, and is the focus of intensive development directed towards photomagnetism in spin-crossover materials.^2–6^ Photophysics was deeply investigated in systems based on iron, ruthenium, cobalt, copper, chromium or manganese.^7–11^ LIESST has been intensely studied in Fe^II^N_6_ spin-crossover materials in which the Fe ion experiences an almost octahedral ligand-field. At low temperature, the ground state is low spin ^1^A_1_ (LS, *S* = 0, t62ge0gL^0^), where L refers to unoccupied ligand orbitals. At high temperatures the high spin state ^5^T_2_ (HS, *S* = 2, t42ge2gL^0^) with a higher entropy is favoured. The photoinduced states are long-lived at low temperature, and Hauser observed that LIESST and reverse-LIESST could be induced by weak continuous laser excitation.^12–14^ Photochromism in crystals associated with conversion between LS and HS states can be directly observed by optical spectroscopy.^15^ LIESST could also induce HS states with structures different from those at thermal equilibrium.^16^ LIESST and reverse-LIESST were transiently generated in solids at high temperatures^17,18^ and observed by pump-probe techniques, including optical and X-ray spectroscopies and X-ray or electron diffraction.^19–25^ The ultrafast molecular expansion during LIESST also drove cooperative, elastic transformations in crystals. It has been shown that more than 7 molecules can undergo switching per photon absorbed.^26,27^ Understanding and controlling LIESST is of great interest and the reason why its basic mechanism has been rigorously investigated for several LS molecules in solution.^28–36^

**Fig. 1:**
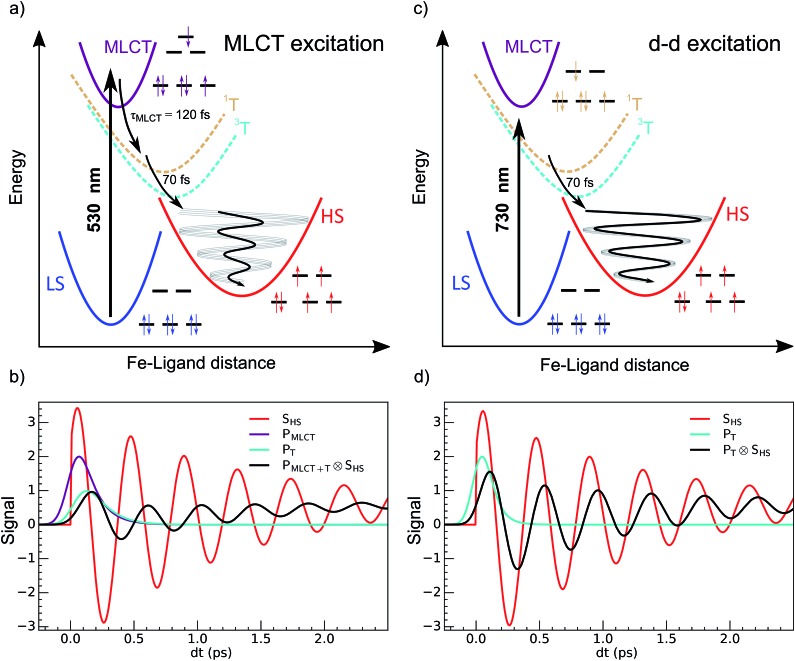
Schematic process of LIESST. (a) For MLCT excitation, the electronic decays towards less bonding ^3^T and HS states increased the equilibrium Fe–ligand distance and launched coherent Fe–L breathing. (b) The weak oscillation of an arbitrary schematic signal (black) due to a change in the Fe–L bond length for MLCT excitation was due to decoherence during the relatively long MLCT-to-HS conversion (populations P_MLCT_(*t*), purple and P_T_(*t*), cyan), compared to the oscillating signal of a single molecule in the HS potential (S_HS_, red). (c) d–d excitation process with fast ^3^T-to-HS conversion. (d) The faster ^3^T decay (population P_T_(*t*), cyan) towards the HS state maintained a significant average structural coherence in the HS potential (black). The exponential growth of the HS population from the MLCT (population P_MLCT_(*t*)), or from the ^3^T intermediate state, led to average coherent oscillating trajectories S_HS_(*t*) ⊗ P_MLCT+T_(*t*) or S_HS_(*t*) ⊗ P_T_(*t*) with a reduced amplitude and apparent phase shift compared to the single trajectory in the HS state S_HS_(*t*).

Recent studies of the LIESST mechanism in Fe^II^ systems upon metal–ligand charge transfer (MLCT) excitation were performed with 30–100 femtosecond time resolution. They revealed key features of the intersystem crossing, mainly in terms of the change in electronic states, as summarized in Fig. 1(a).^5,22,23,37–39^ The MLCT → HS conversion occurred through MLCT → ^3^T and ^3^T → HS conversions of about 120 and 70 fs, respectively. Theoretical electronic-structure calculations used time-dependent approaches to provide intersystem-crossing rates in agreement with these experimental reports.^40^ The HS state was structurally trapped in the HS potential through expansion of the FeN_6_ core. The electronic population of anti-bonding e_g_ orbitals moved the equilibrium molecular structure to longer Fe–L bonds within ∼170 fs. A theoretical study explained how this electron-phonon coupling activated the breathing of the Fe–L bonds,^41^ observed by different techniques sensitive to the change of the ligand field such as XANES^34,38^ or optical spectroscopy.^22,38^ However, the resulting coherent structural dynamics accompanying LIESST is still poorly described in the literature.

**Fig. 2:**
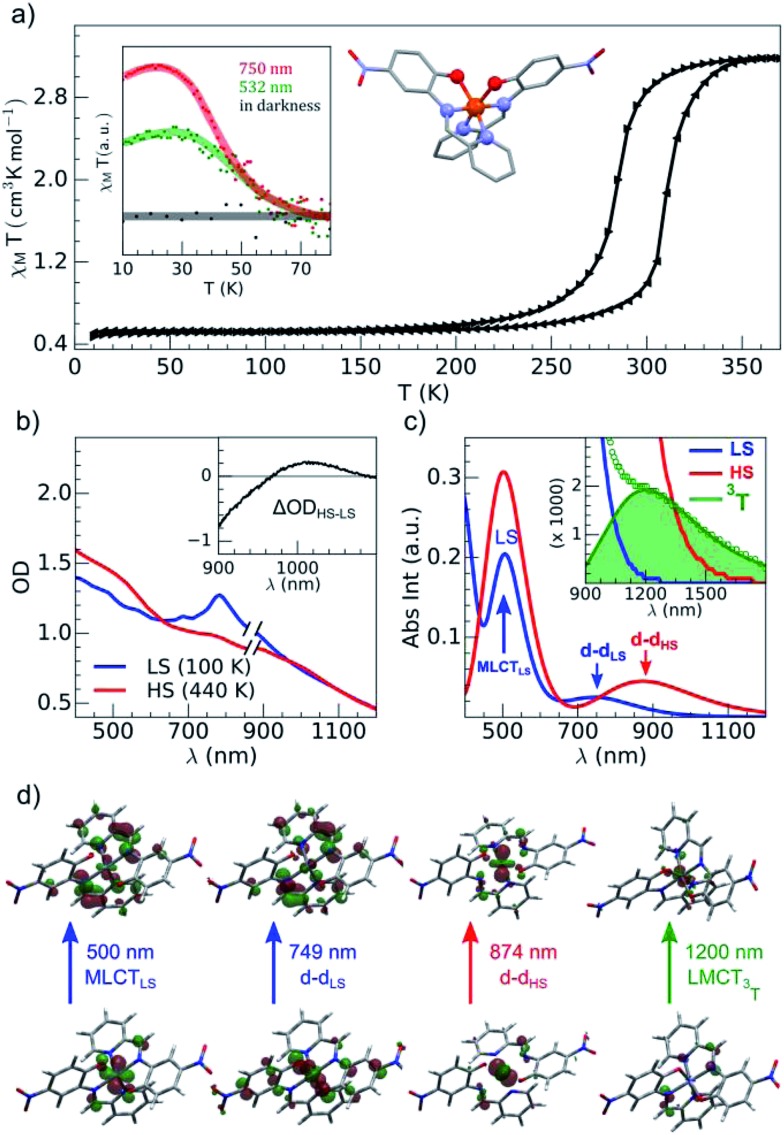
(a) Temperature dependence of *χ*_M_ *T* determined with the sample of Fe(pap-5NO_2_)_2_: thermal cycle (black) and LIESST under photoexcitation at 532 and 750 nm (inset). (b) OD changed between LS and HS states determined with the compound embedded in a polyvinylpyrrolidone (PVP) film. (c) Calculated absorption spectra with main contributions to absorption bands characterized for a single complex for LS (blue), HS (red) and ^3^T (green) states. (d) Characteristic orbitals of hole and particle for LS, HS and T states.

It corresponds to the activation and damping, in the HS potential^17,18,23^ of the average Fe–L distance, which is the main structural reaction coordinate elongating from ∼2.0 (LS) to ∼2.2 Å (HS). Because of the relatively long MLCT and ^3^T decays, the HS population occurred on a timescale approaching the half-period of the molecular breathing,^17,18,22,23^ inducing important structural dephasing. Consequently, compared to a change in signal related to Fe–L elongation like XANES, the amplitude of the signal oscillations was reduced, as illustrated in Fig. 1(b).^38^ The fast damping of the molecular breathing (∼300 fs) was due to dissipation of energy towards other modes such as ligand bending also observed by optical spectroscopy, whereas the vibrational cooling of these photoinduced states typically occurred within 2 ps. The direct photoswitching from *S* = 0 to *S* = 2 was impossible, and the process required electronic intermediates such as ^1,3^MLCT and ^1,3^T. In addition, because of the almost octahedral symmetry of FeN_6_ systems, the d–d photoexcitation (from t_2g_ to e_g_) was very weak or was prevented by much more efficient CT excitations. For this reason, LIESST was mainly investigated at femtosecond timescales with MLCT excitations (t52ge0gL^1^). Hauser used both MLCT and d–d excitation in distinct SCO materials to induce a transient HS state and measure HS → LS relaxation rate dynamics.^42^ Juban and McCusker have shown in Cr-(acac)_3_ that ^4^A_2_ → ^2^E conversion dynamics differed upon ^4^T_2_ or ^4^MLCT excitation.^8^ Herein, we studied LIESST in the Fe^II^ spin-crossover material Fe(pap-5NO_2_)_2_, characterized by an FeN_4_O_2_ core (Fig. 2).^43^ This ligand field, of lower symmetry than that of O_h_, allowed quite intense d–d bands, which we exploited for low-energy photo-excitation. We showed that d–d excitation, associated with shorter electronic intermediates compared to MLCT excitation, allowed a faster LS-to-HS switching and enhanced the coherence of structural dynamics in the HS potential.

## Experimental

### Synthetic procedures

In order to limit the presence of strong light-scattering objects (size ≥ 1 μm) for optical pump-probe studies, the Fe^II^(pap-5NO_2_)_2_ samples were prepared by applying the method described in ref. 44 to the compound synthesis.^43^ The preparation was achieved at –40 °C under inert atmosphere with the Schlenk technique and in presence of PVP ((C_6_H_9_NO)_*n*_, average MW = 40 000 gmol^–1^). A methanolic solution (14 mL, 1 g PVP) of Fe^II^(BF_4_)_2_ (124.8 mg, 0.37 mmol) was added to a methanolic solution (26 mL, 1 g PVP) of Hpap-5NO_2_ (180 mg, 0.74 mmol) previously deprotonated by Et_3_N. After 10 min of stirring, the solution was filtered with a glass microfiber membrane (1 μm pore size), then the filtrate was centrifuged, and the powder of particles was dried under vacuum. Elemental analysis (%) calcd (found) for C_24_H_16_O_6_N_6_Fe (C_6_H_9_NO)_*n*_(H_2_O)_3*n*_ (*n* = 0.7): C, 51.64 (51.40); H, 4.07 (4.06); N, 14.31 (14.2)%. IR: 1667 and 1290 (PVP), 1584, *1452* (C=N and C=C), 1283, *1252* (phenolato group), 1167, *770*, *685* cm^–1^ (in italics, frequencies specific to LS species). The solid, containing traces of PVP 40000, was used without further treatment to avoid the formation of larger particles. For investigating the ultrafast LIESST dynamics, the sample was processed in the form of transparent films on glass substrates by spin-coating a dispersion of particles in *n*-butanol in the presence of PVP.^21^

### Physical measurements

The brown green films of particles appeared to be homogeneous at the sub-millimeter scale. They were characterized by variable-temperature, UV-vis solid-state spectra on a CARY 5000 double-beam spectrophotometer equipped with the Eurolabo variable-temperature cell (21525, KBr windows) and Specac temperature controller (20120). The 100 and 440 K spectra in Fig. 2(b), show all the features previously reported for the high and low-spin phases in the bulk.^43^ Magnetic measurements of the powdered sample characterized a spin transition with a thermal hysteresis centered at 297 K (width Δ*T* = 26 K, Fig. 2(a)) that were comparable to the bulk properties. From the low temperature *χ*_M_ *T* value, a fraction of paramagnetic residue was assigned to the presence of crystalline defects trapped in the HS state (*x*_HS_ ∼0.15). The photomagnetic behavior derived from LIESST at 10 K was previously reported for the bulk with a 750 nm excitation. It was also observed by illuminating the sample (Fig. 2(a)) with a visible (532 nm, 20 mW cm^–2^) and 750 nm (15 mW cm^–2^) excitation (inset Fig. 2(a)). The thermal relaxations towards the LS ground state were completed around 60 K.

### DFT and TD-DFT calculation

In order to better understand the structural, electronic and optical properties of Fe^II^(pap-5NO_2_)_2_, calculations were conducted using the density functional theory (DFT) and time-dependent DFT (TD-DFT). A split-valence Pople basis set of double-zeta quality including polarisation and diffuse functions for non-hydrogen atoms (6-31+G(d))^45^ was used throughout to describe H, C, N and O atoms, while the Los Alamos LANL2 ^46^ effective core potential and corresponding triple-zeta basis set was used to respectively describe the core and valence electrons of Fe^II^. Geometry optimisations of single complexes of Fe(pap-5NO_2_)_2_ in vacuum were conducted using the M06-L^47^ exchange-correlation functional for the different spin states of singlet (LS, *S* = 0), quintet (HS, *S* = 2) and Fe-centred ^3^T (*S* = 1) spin triplet. Essentially, ^3^T should be ^3^Γ due to the C_2_ molecular symmetry, but will be referred to as ^3^T for comparison with FeN_6_ systems. Harmonic vibrational frequencies were obtained both in order to calculate IR properties and to verify that the structures obtained were indeed true minima on the potential energy surface. Electronic excitation energies were calculated using TD-DFT and the PBE0 ^48^ global hybrid exchange-correlation functional. These states were subsequently used to simulate UV-visible absorption spectra for both the LS, HS and ^3^T forms of Fe(pap-5NO_2_)_2_ at their respective equilibrium geometries.

Using the resulting excitation energies and their calculated oscillator strengths, UV-vis absorption spectra were simulated by convoluting with Gaussian functions centred at the calculated wavelength for each transition. The full width at half-maximum value for the Gaussian functions was set to 0.45 eV.

**Fig. 3:**
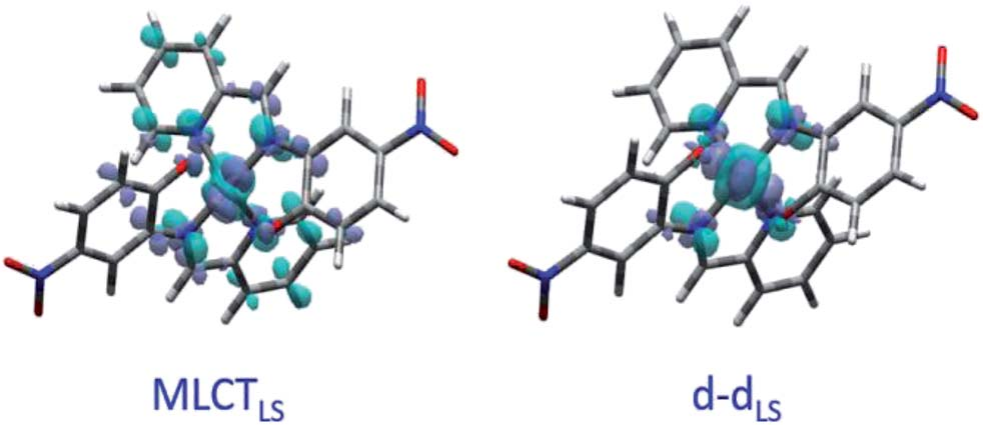
Density difference plots between ground and excited states for MLCT_LS_ and d–d_LS_ excitations. Dark (light) blue zones indicate areas which increased (decreased) in electronic density upon excitation.

Fig. 2(c) shows the vertical absorption spectra calculated for the LS, HS and ^3^T electronic states. Concerning the LS equilibrium geometry, for which singlet-to-singlet excitation energies were determined, two main absorption bands were computed at 500 nm and 749 nm. From inspection of the highest-contributing molecular orbitals involved in these transitions (Fig. 2(d)), it can be seen that, despite contributions from both metal- and ligand-centred orbitals, the transition centred at 500 nm has an MLCT character and is hereafter labelled MLCT_LS_. Similarly, the transition centred at 749 nm has a d–d character and will be referred to as d–d_LS_. Notably, this transition exhibits a small, but non-zero, oscillator strength (0.018 au), emphasizing that the reduced symmetry of the coordination environment from O_h_ for usual FeN_6_ systems to C_2_ for this FeN_4_O_2_ system leads to d–d transitions that are no longer completely forbidden. The character assigned to these transitions was further validated by inspecting plots of the difference in density between the ground and excited states (Fig. 3). In contrast to Fig. 2(d), these plots considered all determinants that form all excited states rather than just those with the largest coefficient.

The d–d transitions for the HS and LS states had a non-negligible intensity with respect to the MLCT transitions due to the reduction of octahedral symmetry. The calculated relative intensities of the HS and LS d–d transitions were consistent since the geometrical distortion from a perfect octahedral symmetry was greater for the HS state than the LS state. More information is provided in ESI.† In addition to singlet-to-singlet (d–d_LS_) excitations, a spin-forbidden, singlet-to-triplet excitation (^3^d–d_LS_) was computed at 823 nm, slightly lower in energy than d–d_LS_, although no information about its intensity could be obtained due to the spin-forbidden nature of the transition. Considering the HS equilibrium geometry, quintet-to-quintet excitations were determined and, as for the LS state, a d–d_HS_ transition centred at 874 nm was identified. Analogously to d–d_LS_, a small but non-zero oscillator strength was determined for d–d_HS_, again demonstrating the effect of the reduced-symmetry (Fig. 2(b and c)).

**Fig. 4:**
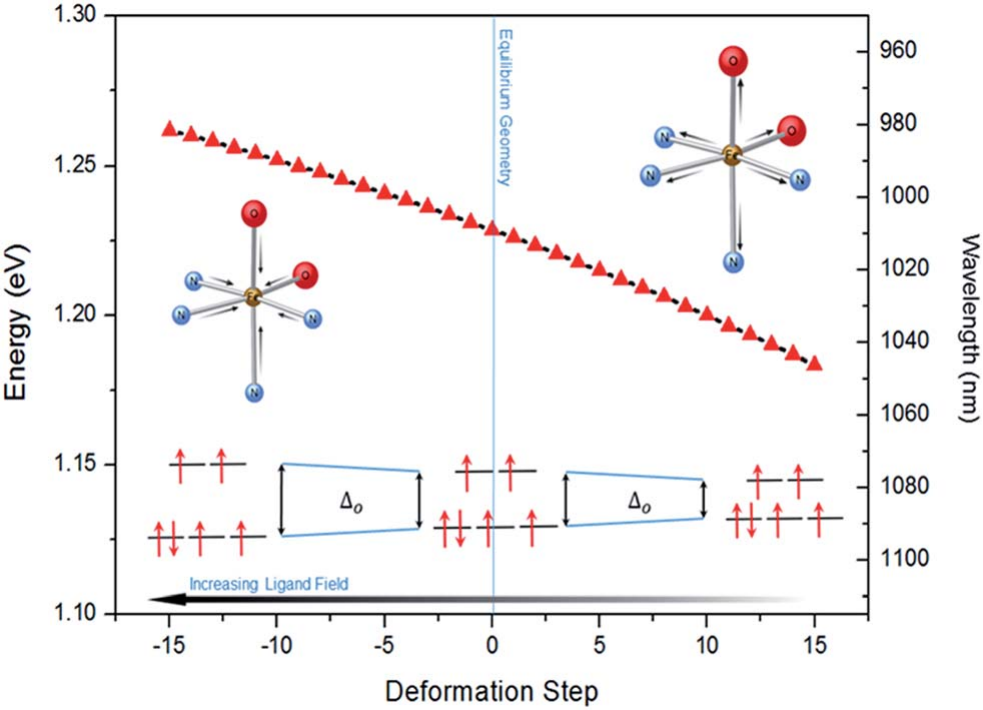
Modulation of *Δ*_0_ (d–d_HS_ splitting) with respect to a harmonic structural deformation, corresponding to the breathing mode of the in-phase stretching of the Fe–N and Fe–O bonds, calculated at 84 cm^–1^. The total deformation lies between changes in Fe–N (axial) from 2.074 to 2.172 Å, Fe–N (plane) from 2.144 to 2.307 Å and Fe–O (plane) from 1.967 to 2.074 Å. On the *x*-axis, 0 corresponds to the equilibrium geometry while negative (positive) values indicate shorter (longer) Fe–L distances.

The lower energy of d–d_HS_ compared with the d–d_LS_ was attributed to the reduced ligand field resulting from the increased Fe–L distances at the HS equilibrium geometry relative to the LS configuration. An additional band centred at around 500 nm was computed, corresponding to a superposition of LMCT and MLCT transitions for the HS state. Considering the vertical absorption spectrum of the ^3^T state, another low-energy absorption band corresponding to an excitation at 1200 nm was found, attributed to LMCT character, and will be referred to as LMCT_3T_. Notably, from the inset in Fig. 2(c), it is shown that this band shares no analogue in the absorption spectra computed for either the LS or HS states. From the vibrational frequency calculations at the HS equilibrium geometry, a mode corresponding to the “breathing” of the Fe–L coordination environment was identified at 84 cm^–1^. This type of breathing mode is characteristic of quasi-octahedral spin crossover compounds. Since this mode corresponds to an oscillation of metal–ligand distances, it can have an observable effect on the d–d_HS_, causing an increase (decrease) in the absorption energy *Δ*_0_, due to the increased (decreased) ligand field imposed by the coordination environment, as observed in other SCO materials.^17,22^ In order to assess the modulation of the ligand field through the d–d_HS_ gap with respect to this breathing, 30 structures were generated as a projection along the normal mode, assuming a harmonic mode, and for each of these, the absorption energy d–d_HS_ was vertically re-computed with TD-DFT (Fig. 4). It can be seen that the increased ligand-field strength with shorter Fe–L distances results in a higher *Δ*_0_, while the opposite was observed for longer Fe–L distances. More details are given in ESI† about the optimised geometrical parameters of the singlet, triplet and quintet states. We also show HOMO, LUMO and SOMO orbitals for each spin state investigated (HS, LS, triplet), as well as the orbitals involved in MLCT, d–d and LMCT transitions with their relative contribution, where possible. Both d–d_LS_ and MLCT_LS_ exhibited contributions from ligand orbitals due to the well-known over-delocalisation of orbitals in DFT. We therefore rationalised the difference between d–d and MLCT transitions from the change (or lack thereof) in d-orbital symmetry upon excitation. We use this theoretical characterisation of the optical fingerprints of the LS, HS and intermediate ^3^T state, in conjunction with femtosecond optical spectroscopy measurements, to ratify different ultrafast LIESST mechanisms observed by pumping particles of LS Fe^II^(pap-5NO_2_)_2_ at two different wavelengths in order to selectively induce LIESST through MLCT or d–d excitation.

### Femtosecond optical pump-probe spectroscopy

For tracking LIESST dynamics in real time we used an optical pump-probe method with 60 fs time resolution (RMS) on LS Fe^II^(pap-5NO_2_)_2_ crystals embedded in a thin PVP film spin-coated on a glass substrate. This allowed easy manipulation of samples with homogeneous dispersion at the sub-millimeter scale.

The pump wavelength was set to 530 nm for MLCT excitation and to 730 nm for d–d excitation. We used similar laser excitation fluence in both cases with 4.0(1) μJ mm^–2^ at 530 nm and 4.2(1) μJ mm^–2^ at 730 nm. Time-resolved OD change measurements were performed at selected wavelengths to track the photoswitching dynamics. The femtosecond optical pump-probe experiments were configured in NIR-transmission geometry with a quasi-collinear configuration of pump and probe beams. The sample temperature was controlled with a liquid nitrogen cryostream set for all experiments at 100 K, where the system is LS and the HS-to-LS back relaxation occurs within less than 1 ms. More details are given in the ESI.†

## Results and discussion

### Photoresponse of LS state to MLCT excitation

**Fig. 5:**
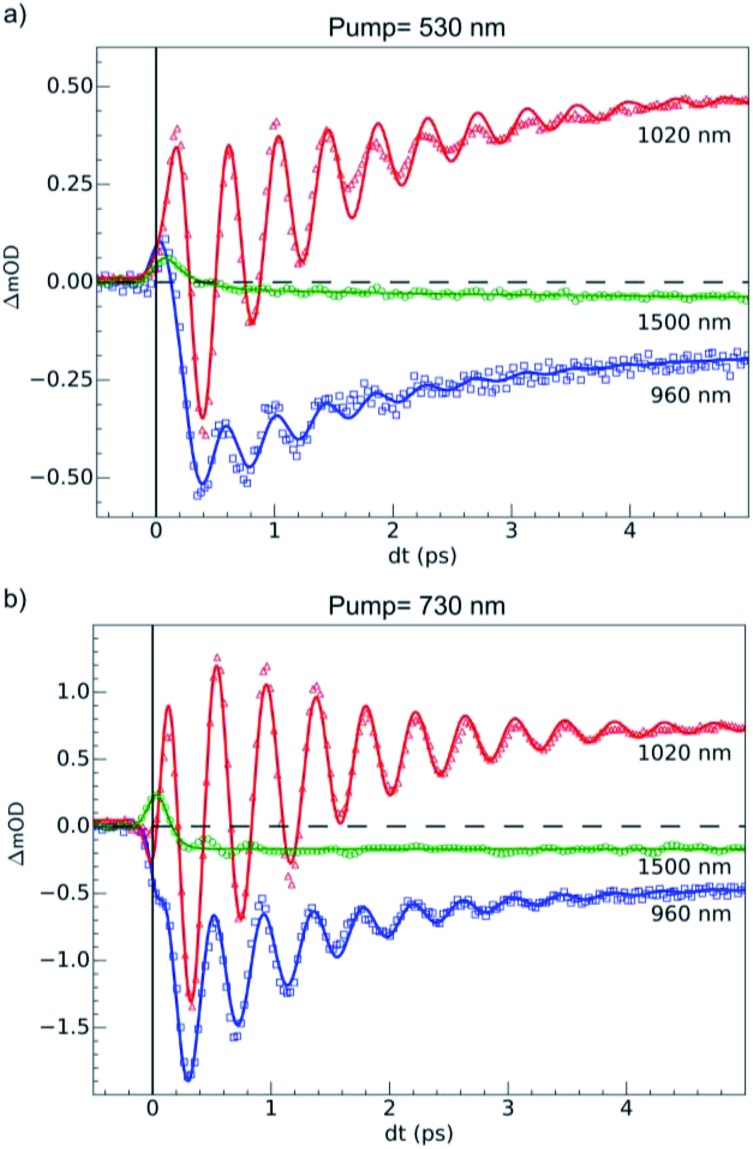
Time scans at 100 K of relative milli-OD change at selected probing wavelengths revealing 80 (3) cm^–1^ oscillations in the 0–5 ps timescale. (a) Dynamics upon MLCT excitation at 530 nm. (b) Dynamics upon d–d excitation at 730 nm. The fits (solid lines) took into account a transient state, coherent oscillation and the 60 fs IRF. We determined values of *τ*_MLCT_ = 188(10) fs, *τ*_dd_ = 70(10) fs and an oscillation frequency *ν*_OSC_ = 80(3) cm^–1^.

OD dynamical time traces probed at 960, 1020, 1500 nm after femtosecond excitation of LS the sample of Fe(pap-5NO_2_)_2_ at 530 nm (*i.e.*, in the MLCT_LS_ band) are shown in Fig. 5(a). The OD increased at 1020 nm and decreased at 960 nm reproducing the optical fingerprints characteristic of the LS → HS switching obtained with steady state spectra (inset Fig. 2(b)). The time traces also indicated an intermediate signal, better observed at 1500 nm within the first hundreds fs, and damped coherent oscillations at 960 and 1020 nm. These features were very similar to the ones reported for ultrafast optical studies of LIESST in Fe^II^N_6_ molecules in solution or in crystals.^17,22,23,39,49^ This was due to the local nature of the process in solids that was reported some years ago^21,26^ and recently confirmed by a direct comparison of the dynamics of Fe(bpy)_3_^2+^ in solution and in crystals.^50^ Time-resolved analysis in spectral regions, where HS and LS species almost equally absorbed (such as 1500 nm), allowed an isolated observation of the dynamics of the intermediate state(s) involved during the spin-state photo-switching. For photoexcitation at 530 nm, such intermediate states (INT) could include the initially photoexcited ^1,3^MLCT state (t52ge0gL^1^) and ^1,3^T (d–d ligand-field states, t52ge1gL^0^) on the pathway towards the HS potential.

Our TD-DFT calculation (Fig. 2) of the triplet spin state indicates a characteristic absorption band around 1200 nm. This band corresponds to an electron transfer from the ligand L to the metal M, and therefore this 1200 nm band of the ^3^T state had an LMCT character and was referred to as LMCT_3T_. Since we could not compute the absorption spectra for the ^1^T and ^1^MLCT states, we used LMCT_3T_ as an approximate global signature of these intermediates using the following qualitative points. Since the orbital energy levels were similar for the ^1^T and ^3^T states, the LMCT_1T_ band of the ^1^T state should also be centred at around 1200 nm. In addition, the initial ^1^MLCT state, resulting from excitation of the LS state at 530 nm, should also have a similar low energy LMCT band due to the partial occupation of its t_2g_-like orbitals. As we see from the LMCT_3T_ state obtained from TD-DFT calculations, these low-energy optical transitions for ^1,3^T and ^1,3^MLCT states have no analogue in either the LS or HS species, even though the tail of the d–d_HS_ band extended up to 1500 nm. Experimentally, the optical absorption of LS and HS states was very low, but quite similar, around 1500 nm due to the broadening of the bands. Data around the isosbestic point at 970 nm (Fig. S2†) were strongly affected by coherent oscillation and vibrational cooling. The transient absorption peak measured at 1500 nm globally included the OD signature of intermediates (^1,3^MLCT and ^1,3^T states) to probe the HS population under MLCT excitation:
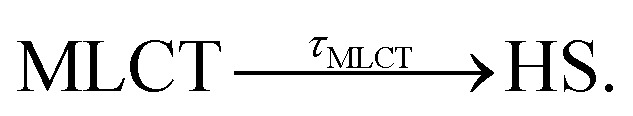


An exponential fit of the data probed at 1500 nm, taking into account our 60 fs RMS instantaneous response function, indicated that the initial MLCT decayed toward the HS state within *τ*_MLCT_ = 188 (10) fs. A slower component *τ*_VC_ = 2.8 (5) ps was also found and attributed to vibrational cooling, as already observed in other SCO materials.^17,18,22,23^ The time-resolved OD data at 960 and 1020 nm showed similar features associated with an initial peak and a slower change accompanied by coherent vibrations. We globally analyzed these data with a phenomenological model. It included the exponential decay of the MLCT state excited at *t* = 0 (and other possible intermediates such as ^1,3^T), populating the final HS state within *τ*_MLCT_ and was accompanied by a damped oscillation. The signal due to the intermediate species was calculated according to the following equation:P_MLCT_(*t*) = IRF(*t*) ⊗ [exp(–*t*/*τ*_MLCT_) × H(*t*)]IRF(*t*) is the Gaussian instrument response function and H(*t*) is the Heaviside function. The signal due to the oscillation in the HS state is described as follows:s_HS_(*t*) = [*A*_HS_ + *A*_OSC_ cos(2π*ν*_osc_ *t*) exp(–*t*/*τ*_osc_) + *A*_VC_ exp(–*t*/*τ*_VC_)] × H(*t*)with amplitudes of the signal related to HS population (*A*_HS_), oscillation (*A*_OSC_) and vibrational cooling (*A*_VC_). Herein, *τ*_OSC_ is the damping of the oscillation with frequency *ν*_osc_. However, since the MLCT state was the source of molecules in the HS state, the observed signal S_HS_(*t*) corresponded to the convolution of S_HS_(*t*) with the MLCT population P_MLCT_(*t*):S_HS_(*t*) = P_MLCT_(*t*) ⊗ s_HS_(*t*)as schematically shown in Fig. 1(b).

For the fit of the data probed at 960 and 1020 nm (Fig. 5(a)), the physical parameters *ν*_osc_ = 80(3) cm^–1^ and *τ*_MLCT_ = 188(12) fs were the same for the different probing wavelengths. The 188 fs decay from the MLCT to HS state was in good agreement with the independent fit at 1500 nm. However, the vibrational cooling timescale *τ*_VC_ and the oscillation damping *τ*_osc_ were different for each wavelength. It is well known that these timescales depend on the spectral region. The damping constant was *τ*_osc_ = 1.001 (0.05) ps at 1020 nm and 1.208(0.02) ps at 960 nm, and the vibrational cooling occurred within *τ*_VC_ = 1.56(0.05) ps at 1020 nm and 1.68(0.05) ps at 960 nm. Again, these timescales were similar to the values reported for other systems presenting LIESST in solids or in solution.^5,22,23,37–39^

The 80(3) cm^–1^ oscillations were observed, once the HS reached, for OD time traces at 960 and 1020 nm (*i.e.*, close to the d–d_HS_ band (Fig. 2(c)). These were associated with a strong modulation of the ligand field *Δ*_0_ by molecular breathing (Fig. 4). Similar findings were also reported in other SCO crystals, and this oscillation probed around the d–d_HS_ transition was attributed to the breathing of the ligand field.^12,22,23^ For FeN_6_ systems, this was the main reaction coordinate between the LS and HS structures, which corresponded to the in-phase elongation of the Fe–N bond lengths. Our DFT study on Fe^II^(pap-5NO_2_)_2_ revealed a breathing mode in the HS state at 84 cm^–1^. This completely symmetric vibration mainly involved the FeN_4_O_2_ core and also corresponded to in phase stretching of Fe–N and Fe–O bonds as shown in Fig. 4. These results indicated that the physical picture for MLCT-induced LIESST in Fe(pap-5NO_2_)_2_, driven by the MLCT decay towards lower states activating and damping molecular breathing, was similar to the one theoretically and experimentally reported for FeN_6_ systems (Fig. 1(a)).^17,18,22,23,38,41^ The coherent oscillations observed during LIESST were sometimes attributed to an impulsive Raman process activating the LS breathing mode. This possibility can be excluded because the LS breathing frequency was significantly higher (146 cm^–1^). In addition, an impulsive process would induce sine-like oscillations around zero OD change, whereas the band probed at 1020 nm, where oscillations are stronger, is sensitive to the formation of the HS state.

### Photoresponse of LS state to d–d excitation

Additional measurements were also performed at 100 K in the LS state upon d–d excitation at 730 nm generating ligand-field states (^1,3^T, t52ge1gL^0^), with experimental conditions similar to the ones used for MLCT excitation. Time-resolved OD changes for the different probing wavelengths are shown in Fig. 5(b). At 5 ps, the OD increase (decrease) at 1020 nm (960 nm) and a weak change at 1500 nm were characteristic of the formation of the HS state and similar to the changes observed upon MLCT excitation at 530 nm. However, the OD change was larger for d–d excitation when a similar laser fluence was used. For example, at 1020 nm, the OD increased by 0.48 for a 530 nm excitation and by 0.72 for a 730 nm excitation (Fig. 5(b)). In SCO materials, the quantum efficiency has been known to be close to unity, and the OD linearly changed with the number of photons in such low fluence regimes.^26^ The ratio of the number of photons contained in the pump laser pulses of 4.2(1) μJ mm^–2^ at 730 nm and 4.0(1) μJ mm^–2^ at 530 nm was *R*_ph_ ≈ 1.45(5). This ratio was close to the ratio of the OD change at 1020 nm between 730 nm and 530 nm excitations: *R*_OD_ = 1.5. These results indicated that d–d and MLCT excitations generated the final HS state with a very similar quantum yield. The comparison with data at 960 nm yielded a value on the same order, but was more difficult to analyze because 960 nm was closer to the isosbestic point, known to shift by lattice pressure and heating following femtosecond excitation.^21^ Additional data are presented in Fig. S2,† where the in-phase oscillations of time traces recorded at different probing wavelengths are characteristic of the global oscillation in wavelength of the d–d_HS_ band with Fe–L oscillation, as discussed in Fig. 4.

Data at 1020 nm, around the maximum of OD change (Fig. 2(b)), were not sensitive to this effect and better suited to observe quantum yield. Around *t* = 0 ps at a 960 nm probing wavelength, the OD decreased for the 730 nm d–d excitation, contrary to the 530 nm MLCT excitation. In addition, the OD changes observed around *t* = 0 ps at 1020 and 1500 nm probe for 730 and 530 nm excitations were similar, but with different relative amplitudes. These observations indicated two different initial states with distinctive spectroscopic signatures, in agreement with TD-DFT calculations of the LS state (Fig. 2(d)).

We identified the initial state as a ^1,3^MLCT upon 530 nm excitation and as a ^1,3^T state upon 730 nm excitation. Our calculations showed that photoexcitation of the d–d_LS_ band could induce ^1^T spin-allowed or ^3^T spin-forbidden states, but the relative weights of these could not be calculated, and two possible pathways may (co)exist, LS → ^1^T → ^3^T → HS and/or LS → ^3^T → HS. Since Sousa *et al.* calculated ^1^T → ^3^T inter-system crossing rates in the 400–2700 fs timescale and ^3^T → HS in the 60 fs range,^40^ it is possible that the d–d_LS_ photoexcitation mainly induced the ^3^T spin-forbidden states. Femtosecond X-ray fluorescence measurements, as performed by K. Gaffney on Fe(bpy)_3_^2+^,^37^ may be helpful in the future to discriminate the nature of the initial photo-excited state. In order to fit the time evolution of the optical fingerprints of the different states, we used the kinetic model described for MLCT excitation, now including the exponential decay of the photoinduced T state towards the final HS state as the initial process:
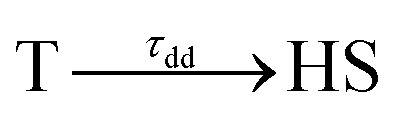


The fit of the data (Fig. 5(b)) indicated that the HS state was populated after d–d excitation within *τ*_dd_ = 70(10) fs. The process was also accompanied by a coherent oscillation, with frequency *ν*_osc_ = 80(3) cm^–1^ and damping constant *τ*_osc_ = 1.21(0.02) ps at 1020 nm and 1.00(0.02) ps at 960 nm. The vibrational cooling occurred within *τ*_VC_ = 1.33(0.04) ps at 1020 nm and 1.49(0.02) ps at 960 nm. The fact that the frequency of the oscillation corresponded to the one observed after MLCT (530 nm) excitation confirmed that the final photoinduced state was the same under d–d (730 nm) excitation, as already indicated by the similar OD changes and photomagnetism. The main difference compared to the MLCT excitation was the faster population of the HS state, since the number of intermediates was reduced in the case of d–d excitation compared to MLCT excitation.

**Fig. 6:**
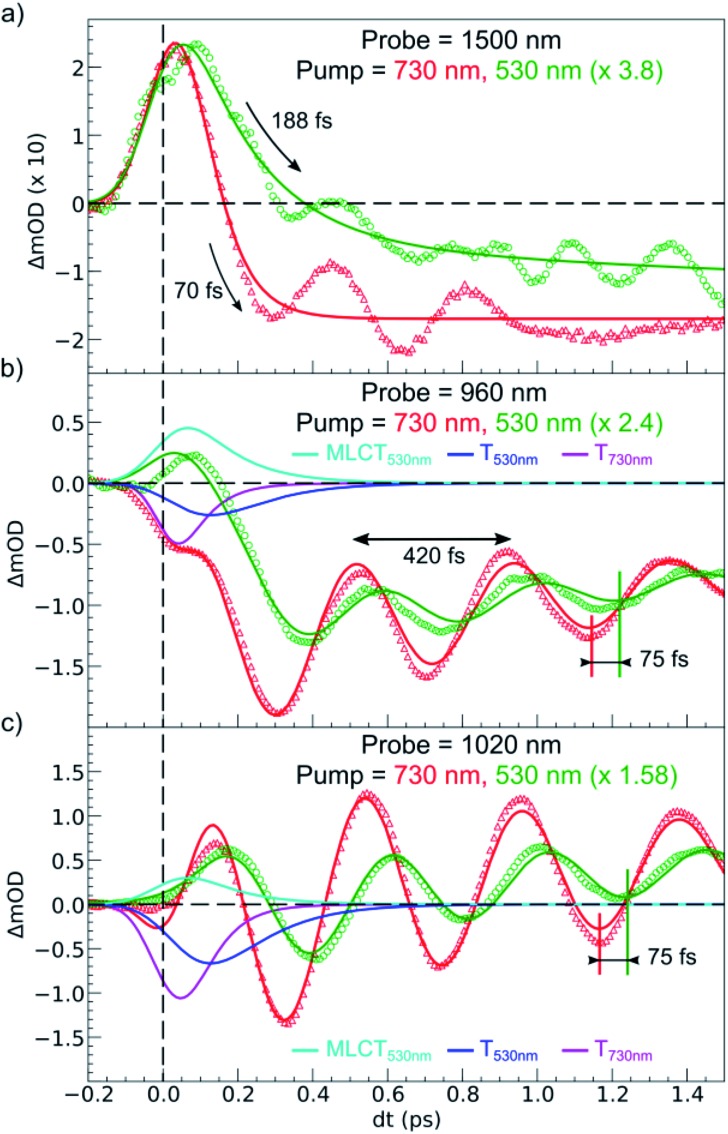
Comparison of short time scans of relative OD changes at selected probing wavelengths upon MLCT_LS_ excitation at 530 nm and d–d_LS_ excitation at 730 nm. The fit for the 530 nm excitation includes MLCT and T intermediates, whereas the fit for 730 nm includes T only.

Fig. 6 compares the OD changes for the different probing wavelengths on short timescales. The amplitudes of the changes were scaled for clarity. The OD peak probed at 1500 nm clearly appeared to be longer around *t* = 0 under MLCT than under d–d excitation. Regarding the oscillating components, there were two main differences between the two excitation processes. There was a phase shift, of about 75 fs, between the oscillation observed after MLCT and d–d excitation. Moreover, the relative amplitude of the oscillation, with respect to the OD change between LS and HS states, was larger under d–d excitation than under MLCT excitation.

These features are explained very well by our model, which describes the signal as an initial photoexcited state, (MLCT or T) which exponentially populates the HS potential where molecules undergo damped oscillations. Both effects result from the shorter decay time *τ*_dd_ = 75(10) fs under d–d excitation than *τ*_MLCT_ = 188(10) fs under MLCT excitation. The T state, with an electronic structure of t52ge1gL^0^, is a natural intermediate between MLCT (t52ge0gL^1^) and HS (t42ge2gL^0^) states, which was observed by X-ray spectroscopy in Fe(bpy)_3_^2+^.^37^ We therefore performed another fit of the data in Fig. 6 to describe the consecutive decays under MLCT excitation:
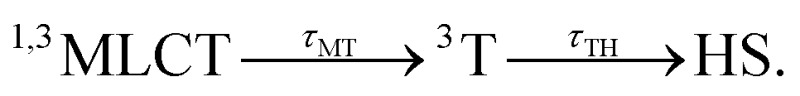


We independently found *τ*_TH_ under d–d excitation as *τ*_TH_ = *τ*_dd_ = 70(10) fs. Therefore, in the fit presented in Fig. 6, we only refined the ^1,3^MLCT → T decay time constant *τ*_MT_ = 120(10) fs. The faster HS population under d–d excitation maintained coherence (amplitude of the oscillation) and more rapidly initiated HS state oscillation, as illustrated by the oscillation phase shift in time. This was due to the fact that the HS population was much faster than the 210 fs half oscillation period, which was the upper limit on the timescale to maintain oscillation coherence in the HS state. Under MLCT excitation, the overall *τ*_MLCT_ = 188(10) fs HS population approached the half oscillation period and was responsible for decoherence during the long MLCT-to-HS population, reducing the amplitude of the oscillation. These effects are summarized in Fig. 1(b and d).

## Conclusions

This study compared two possible LIESST pathways from LS to HS states under d–d or MLCT photoexcitations. This was made possible by chemical engineering through a ligand design with low symmetry that allowed intense d–d bands while maintaining the spin-crossover and LIESST properties. The sequences MLCT → 3T within 120 fs and 3T → HS within 70 fs were very similar for FeN_6_ and the lower symmetry FeN_4_O_2_ system investigated here. LIESST was associated with the fast activation of the molecular breathing mode since anti-bonding “e_g_-like” orbitals were populated, and hence increased the equilibrium Fe–L distance. This structural trapping of the system in the HS potential was similar to other systems.^17,18,22,23^ Therefore, the short-lived intermediates were not thermally equilibrated states, since their population had a shorter lifetime than the oscillation period in the potential. They corresponded to an evolution of hot states within the manifold of electronic states involved in the process, where the coherent structural dynamics moved the system between different electronic potentials, on a timescale where wave functions of atomic nuclei and electrons are difficult to separate. The resulting physical picture was consistent with the theoretical model introduced by van Veenendaal.^41^ In addition, the speed of the structural trapping through Fe–L elongation was limited by the decay of the MLCT towards the T state, where antibonding orbitals were populated since the initial ^1^MLCT state had on equally bonding character as the initial LS state. The molecular expansion only began once the T state was reached, which took about 120(10) fs, before decaying towards the final HS state within 70(10) fs. The d–d excitation process instantaneously populated antibonding orbitals, which launched the Fe–L expansion and moved the system towards the HS potential. The HS state was then reached within 70(10) fs. Herein, we could accurately study the coherent structural dynamics during LIESST through the intense d–d_HS_ band in the FeN_4_O_2_ system, strongly modulated by the Fe–L distance. By reducing the lifetime of intermediates compared to MLCT excitation, d–d excitation allowed a faster LIESST and preserved structural breathing coherence since this molecular reaction coordinate was activated and damped to trap the HS state.
