# Supplementary material for: Comparison of structural dynamics and coherence of d–d and MLCT light-induced spin state trapping
Source: Chem Sci. 2017 Apr 24;8(7):4978–86. doi: 10.1039/c6sc05624e (PMC5607858; doi:10.1039/c6sc05624e)
Supplement: Supplementary file 1 [file SC-008-C6SC05624E-s001.pdf]

Electronic Supplementary Material (ESI) for Chemical Science.
This journal is © The Royal Society of Chemistry 2017

Supplementary Information

List of Supplementary information

1 Optical pump-probe spectroscopy

1.1 Cross-phase modulation.

1.2 Time scan at different probing wavelengths

2 DFT and TD-DFT calculation

2.1 Geometrical parameters

2.2 Frontier orbitals of the different electronic states

1 Optical pump-probe spectroscopy.

1.1 Cross-phase modulation. For each experiment on the sample deposited on the glass substrate, we checked possible cross-phase modulation (CPM) signal, by performing measurements with the same conditions (overlap, position, lasers power) on the glass substrate to find accurately the time zero (pum-probe overlap, Fig. S1). The amplitude of the CPM is negligible comparing to the real signal from the sample and we sometime had to increase the laser power by a factor ×2.5-12 to observe weak CPM.

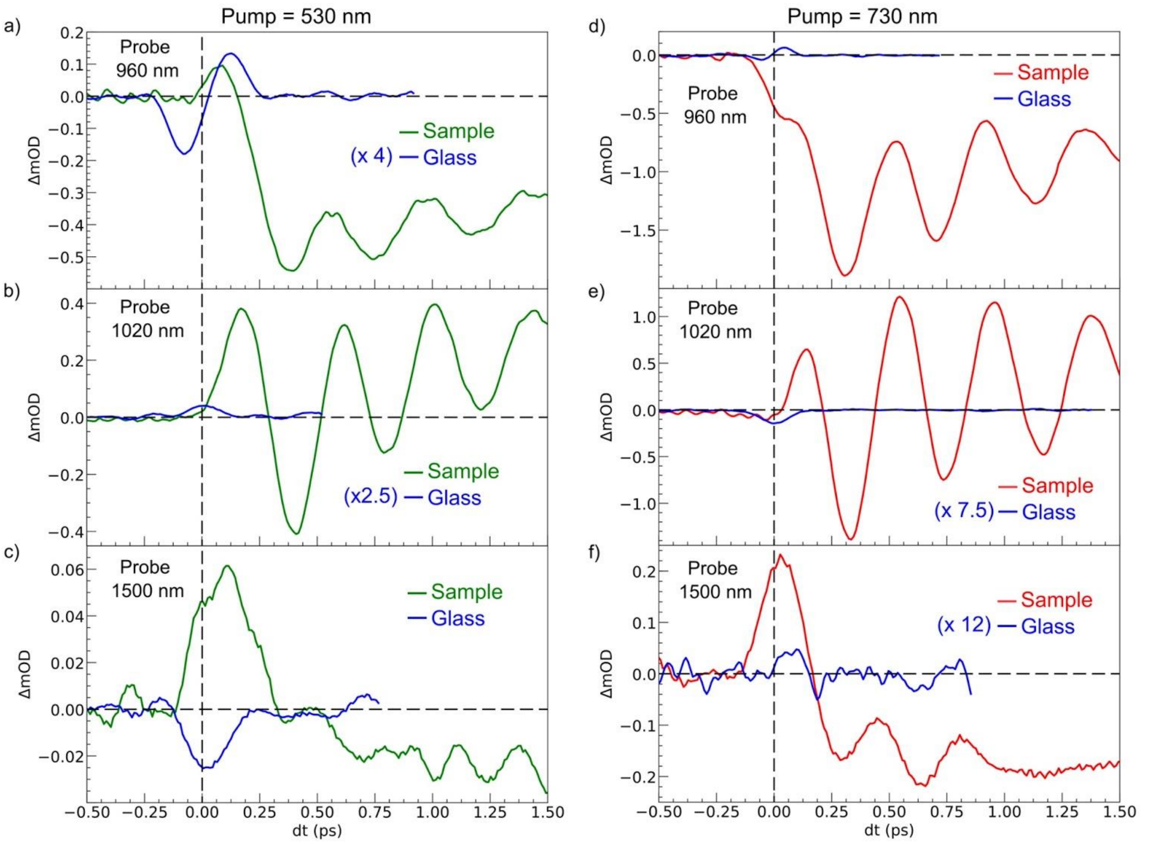

Figure S1. Timescan with and without the sample on the glass, showing weak cross-phase modulation. The laser power increase for detecting CPM on the glass is given in the figures.

1.2 Time scan at different probing wavelengths. We performed additional pump-probe measurements for pump wavelength set to 730 nm at different probing wavelengths. The data shown in Fig. S2 have in-phase oscillations, characteristic of the global shift of the d-d_{HS} band with Fe-L change. Characteristic features of these time traces are similar to the ones presented in the manuscript (frequency of the mode, τ_{MLCT}, ...). Time traces at 980 and 1000 nm show signatures of the intermediate state, but are also affected by the proximity of the isobestic point (970 nm, Figure 2(b)), which shifts in time during the vibrational cooling, as observed in other SCO crystals. The signal of these time traces therefore changes signs between 100-500 fs (hot HS state) and after 5 ps (vibrationally cool HS state). Below 940 nm oscillations are more difficult to observe.

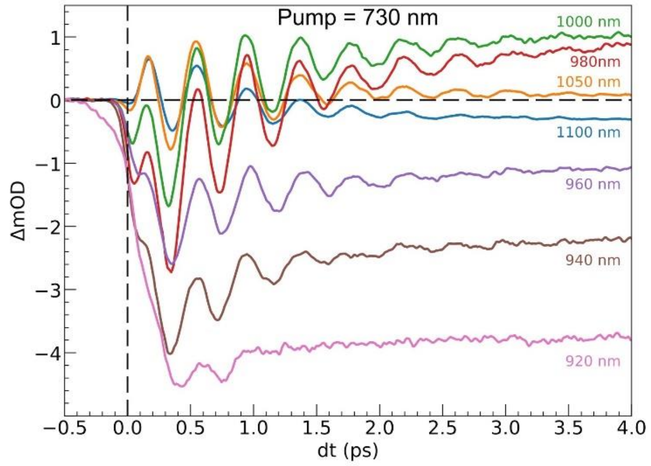

Figure S2. Time scans of relative milli-OD change at in the 920-1100 nm range revealing 80 (3) cm-1 oscillations, upon d-d excitation at 730 nm.

2 DFT and TD-DFT calculation

2.1 Geometrical parameters

Figure S3. Schematic of bond lengths and angles outlines in tables S1-S2.

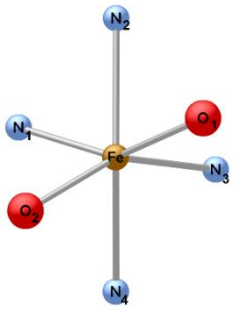

Table S1. Geometrical parameters of Fe-L core. Fe-L bond lengths (in Å) and angles (in °) are given.

Parameter	Singlet	Triplet	Quintet
$Fe - N_1$	1.954	1.965	2.184
$Fe - N_2$	1.883	1.905	2.091
$Fe - N_3$	1.955	1.095	2.184
$Fe - N_4$	1.883	1.905	2.091
$Fe - O_1$	1.971	1.881	2.066
$Fe - O_2$	1.971	1.881	2.066
$\angle N_1 - Fe - N_2$	81.94	82.07	75.57
$\angle N_1 - Fe - N_3$	90.93	88.41	90.30
$\angle N_1 - Fe - N_4$	97.44	101.66	95.49
$\angle N_1 - Fe - O_1$	165.82	166.59	152.82
$\angle N_1 - Fe - O_2$	92.69	88.83	93.44
$\angle N_2 - Fe - N_3$	97.44	101.66	95.49
$\angle N_2 - Fe - N_4$	179.13	174.86	167.50
$\angle N_2 - Fe - O_1$	83.99	85.27	77.28
$\angle N_2 - Fe - O_2$	96.63	91.25	111.51

Table S2. Deviation of each spin species from a perfect octahedral environment (in terms of angles). Each Number represents the absolute value of the difference between the calculated angle and 90°or 180° where appropriate.

Parameter	Δ Singlet	Δ Triplet	Δ Quintet
∠ N_1 – Fe – N_2	8.06	7.93	14.43
∠ N_1 – Fe – N_3	0.93	1.59	0.3
∠ N_1 – Fe – N_4	7.44	11.66	5.49
∠ N_1 – Fe – O_1	14.18	13.41	27.18
∠ N_1 – Fe – O_2	2.69	1.17	3.44
∠ N_2 – Fe – N_3	7.44	11.66	5.49
∠ N_2 – Fe – N_4	0.87	5.14	12.5
∠ N_2 – Fe – O_1	6.01	4.73	12.72
∠ N_2 – Fe – O_2	6.63	1.25	21.51
MEAN	6.03	6.50	11.45

2.2 Frontier orbitals

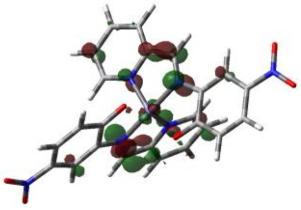

LUMO

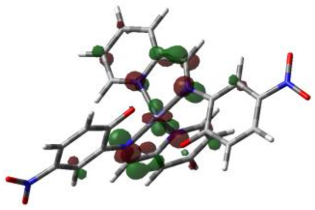

LUMO+1

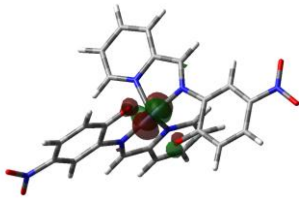

HOMO-2

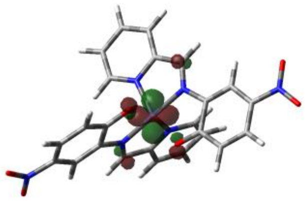

HOMO-1

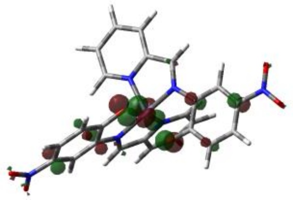

HOMO

Wavelength<br>(nm)	Osc. Strength<br>(au)	Orbitals<br>(% contribution)	Character
749	0.009	HOMO – LUMO+1 (87%)	d-d
500	0.090	HOMO-1 – LUMO+1 (34%)<br>HOMO-2 – LUMO (58%)	MLCT

Figure S4. Top: Frontier orbitals for the low-spin (singlet) species of Fe(pap-$5NO_2$)$_2$. Iso-contour value is 0.06. Bottom: MLCT and d-d transition information with the percentage contribution of significant transitions in the CI expansion, derived from the CI coefficients.

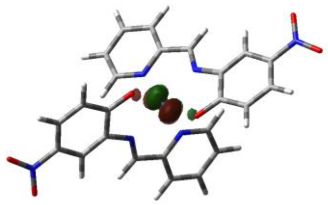

SOMO

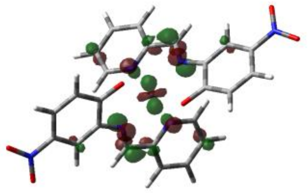

SOMO-1

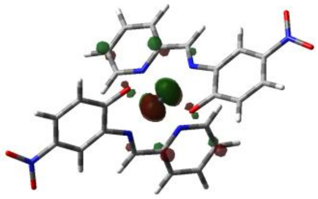

SOMO-2

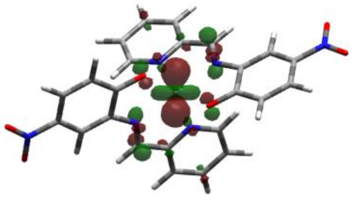

SOMO-3

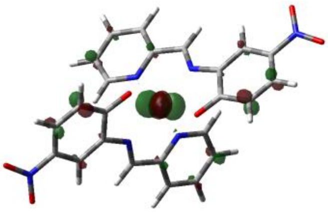

HOMO

Wavelength (nm)	Osc. Strength (au)	Orbitals	Character
874	0.018	HOMO – SOMO-3	d-d

Figure S5. Top: Frontier orbitals for the high-spin (quintet) species of Fe(pap-5NO_2)$_2$. Iso-contour value is 0.06. Botttom: d-d transition information.

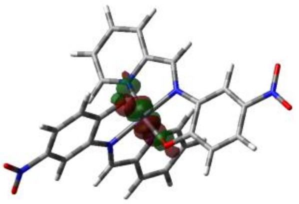

LUMO

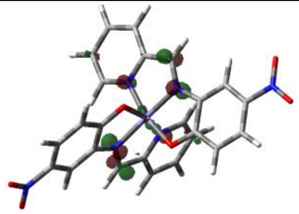

SOMO

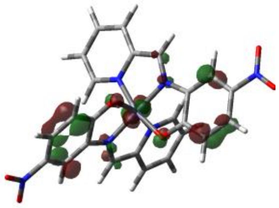

SOMO-1

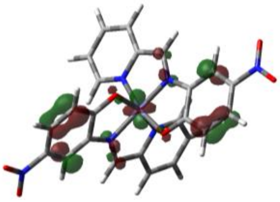

HOMO

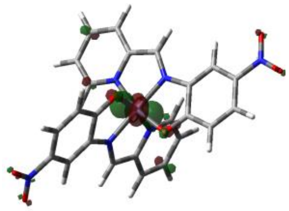

HOMO-1

Wavelength (nm)	Osc. Strength (au)	Orbitals	Character
1200	0.010	SOMO – LUMO	LMCT

Figure S6. Top: Frontier orbitals for the intermediate-spin (triplet) species of Fe(pap-5NO_2)$_2$. Iso-contour value is 0.06. Botttom: LMCT transition information
